# A Case Report of Fournier’s Gangrene

**DOI:** 10.21980/J8Z356

**Published:** 2022-04-15

**Authors:** Huy Alex Duong, Mark Slader, Jana Florian, Jonathan Smart

**Affiliations:** * University of California, Irvine, Department of Emergency Medicine, Orange, CA

## Abstract

Early identification and intervention of Fournier’s gangrene in the Emergency Department (ED) requires a high index of suspicion and is critical in improved patient outcomes. We present a case of a 64-year-old male with two months of progressively worsening buttock and rectal pain found to have extensive black eschar and ecchymosis on exam. In addition, this patient displayed marked leukocytosis, lactic acidosis, and elevated inflammatory markers. A preoperative computed tomography (CT) scan of the abdomen and pelvis revealed multiple perirectal abscesses and subcutaneous fluid and gas in the perineum and scrotum concerning for Fournier’s Gangrene. He was started on broad spectrum antibiotics while in the ED and taken to the operating room for urgent surgical debridement with clinical improvement and was discharged home on day nine. This case report reviews the clinical presentation and interventional modalities, and aims to provide new images to better help visualize a diagnosis of Fournier’s Gangrene.

**Topics:**

Fournier’s gangrene, necrotizing soft tissue infection, necrotizing fasciitis.

**Figure:**
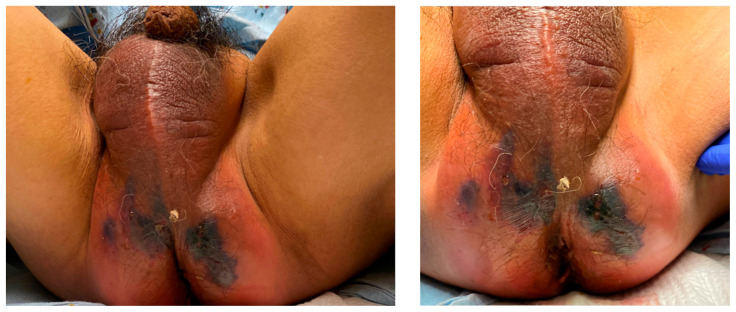


**Figure:**
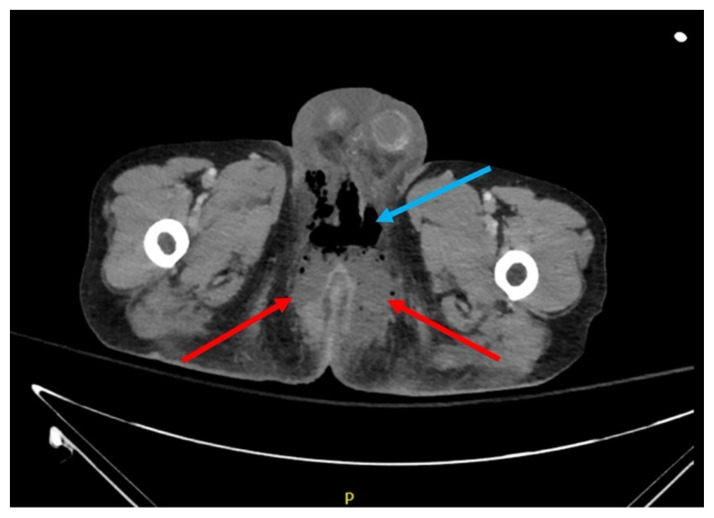


## Brief introduction

Fournier’s gangrene is a specific subclassification of necrotizing soft tissue infections (NSTI) affecting the external genitalia, perineum, or perianal regions and constitutes a surgical emergency which must be rapidly recognized on exam.^1^ Current research indicates that Fournier’s Gangrene is most commonly caused by polymicrobial organisms infecting the subcutaneous tissue with subsequent inflammation followed by eventual necrosis. Common sources of infection include the urinary tract, colorectal, and anorectal regions.^2^,^3^,^4^ Certain risk factors have been shown to have some association with developing Fournier’s gangrene such as diabetes mellitus, obesity, and immunosuppression.^5^ While initially believed to primarily affect men, recent studies have noted involvement of children and women as well.^2^,^6^ Early symptoms may include fever, swelling, and erythema. This nonspecific presentation may allow the condition to be mistaken for cellulitis or abscess, hindering rapid diagnosis. Classically taught symptoms of NSTI such as crepitus, skin necrosis, ecchymosis or gas on radiographic imaging are generally late-onset findings.^7^ Current practice guidelines include prompt initiation of broad-spectrum antibiotic therapy and urgent surgical debridement. Even with ideal care, mortality rates remain high and are often accompanied by prolonged hospital courses. 3,8

Fournier’s gangrene is a rare diagnosis that requires a high index of suspicion with rapid recognition being critical to optimize patient outcomes. Here, we describe a case of a 64-year-old male diagnosed with Fournier’s gangrene following two months of rectal pain. We further discuss clinical findings, workup, current recommendations for treatment, and provide new images to help better visualize this rare but emergent infection. Patient-written consent for medical photography was obtained during the patient’s admission.

## Presenting concerns and clinical findings

A 64-year-old male with a past medical history of daily tobacco use presented to the ED complaining of severe pain of the bilateral buttocks and rectum for the previous two months. Describing this pain as sharp, he explained that it has progressively worsened since its onset. His pain worsened during bowel movements and with sitting. The patient also endorsed associated diarrhea, intermittent hematochezia, and melena as well as unintentional weight loss of 12 pounds over two months. He denied fevers, chills, nausea, vomiting, abdominal pain, HIV, diabetes, steroid use, intravenous drug use, and family history of malignancy. Upon arrival to the ED, the patient was afebrile (99° F), tachycardic with a heart rate of 130 per minute, blood pressure of 140/67 mmHg, respiratory rate of 18 per minute, and pulse oximetry of 100% on room air.

## Significant findings

Physical exam revealed a comfortable-appearing male patient with tachycardia and a regular cardiac rhythm. The genitourinary exam indicated significant erythema and fluctuance of the bilateral lower buttocks with extension to the perineum. Black eschar and ecchymosis were also noted at the perineum. There was significant tenderness to palpation that extended beyond the borders of erythema. There was no palpable crepitus on initial examination. Physical exam was otherwise unremarkable.

The patient’s white blood cell count (WBC: 42.9 thous/mcl) was significantly elevated, hemoglobin (Hgb: 9.9 g/dL) was low, serum lactate (3.1 mmol/L) was elevated, and serum pH (7.34) was low. Additionally, the patient’s serum sodium was low (134 mmol/L) while serum C-reactive protein (CRP: 33.4 mg/dL) and erythrocyte sedimentation rate (ESR: 69 mm/hr) were elevated. When combining the patient’s tachycardia, physical exam findings concerning for infection, leukocytosis, and lactic acidosis, the patient met criteria for severe sepsis by *Sepsis-3 Consensus Definitions*.^9^ While pending an OR being prepared, a CT scan of the abdomen and pelvis revealed multiple perirectal abscesses, left worse than right, above and below the levators, with fistulation through the left levator ani musculature (red arrows). There was also extensive subcutaneous fluid and gas in the soft tissues of the perineum and scrotum (blue arrow). The radiologist noted the subcutaneous gas may be due to Fournier’s gangrene or air tracking from the rectal lumen. Additionally, a CT scan of the chest indicated there were multiple scattered, partially calcified and noncalcified pulmonary parenchymal and subpleural nodules with concern for metastatic nodules versus post infectious granulomas.

In addition, the patient’s leukocytosis, moderate anemia, hyponatremia, and elevated inflammatory markers combined to result in a Laboratory Risk Indicator for Necrotizing Fasciitis (LRINEC) score of 10.^10^

## Patient course

The patient’s initial presentation was concerning for Fournier’s gangrene with severe sepsis, which required prompt medical and surgical intervention. Shortly after evaluation, the patient was started on broad-spectrum antibiotics (vancomycin, piperacillin/tazobactam, and clindamycin), intravenous normal saline, and the emergency general surgery (EGS) team was STAT paged to evaluate.

Given the patient’s presentation at the time alongside the previously noted CT findings, the patient was emergently scheduled for perineal debridement, abscess drainage, and drain placement by EGS in conjunction with the colorectal and urology surgical teams. He was admitted to the intensive care unit (ICU) following debridement. On the following day, the patient was taken back to the operating room (OR) for a diagnostic laparoscopy, biopsy of a peritoneal nodule, colostomy construction, further perineal wound debridement, flexible sigmoidoscopy, and endoscopic biopsy of rectal mass.

Pathology evaluation of the rectal mass revealed invasive and moderately differentiated adenocarcinoma consistent with colorectal origin. Evaluation of the peritoneal nodule indicated fat necrosis with fibrocalcific change that was negative for carcinoma.

He was transferred to a step-down unit following his second surgical procedure. The patient was clinically improving until hospital admission day 6 when he started having nausea, vomiting, bloated abdominal pain, and decreased ostomy output. An X-ray of the upper abdomen revealed gaseous distention of the small and large intestine indicating a likely postoperative ileus. A CT scan of the abdomen and pelvis again showed diffuse gaseous distention of the small and large intestine with concern for a postoperative ileus versus an obstructive process at the colostomy. The opening of the colostomy was widened the following day, which resulted in 1450 cc of fecal output. The patient’s pain progressively improved following the increased stool output.

The patient was discharged home on hospital day nine after wound care teaching, enoxaparin training, and home health medical care were established. He was discharged with oral antibiotics, stool softeners, and pain medication. At the patient’s subsequent postoperative visits, his pain was subjectively improved, and his perineal wound healed without further signs of infection.

## Discussion

Fournier’s gangrene is a rapidly progressive form of necrotizing infection involving the external genitalia and often involves the perineum.^1^ Infection initially occurs at the level of the hypodermis and can occur due to any disruption to the epithelial surface ranging from traumatic injury, abscesses and even urinary tract infections and stones.^7^ Common sources include the genitourinary tract, anorectal region, or involvement of any part of the external genitalia or perineum. Bacteria then break down surrounding tissue leading to further spread and eventual necrosis and gangrene. Some studies postulate that the tissue hypoxia and resulting necrosis and gangrene may in part be due to thrombosis in addition to toxin release and tissue breakdown.^11^ The most common microorganisms responsible for Fournier’s are typically polymicrobial in nature including both aerobes and anaerobes. This is followed by monomicrobial organisms such as *Escherichia coli* and *Streptococcus*.^3^,^4^ Several conditions have been found to be associated with Fournier’s gangrene including older age, diabetes, alcoholism, immunosuppression (ie, HIV), and obesity.^5^ Patients typically present early with non-specific findings akin to cellulitis with surrounding erythema and induration. Progression to necrosis and classic findings of Fournier’s gangrene presents later and patients often present to the hospital severely ill or septic.

Current mortality rates are variable. An evaluation of 1726 cases by Eke et al. described an overall mortality rate of 16% with causes of death including severe sepsis, coagulopathy, acute renal failure, and multi-organ failure.^2^ Other epidemiologic studies have demonstrated overall mortality rates as low as 7.5% though these do note that specific hospitals have demonstrated a mortality rate as high as 88% when caring for Fournier’s patients. These differences may reflect hospital exposure to Fournier’s cases and their ability to rapidly recognize and treat the condition effectively. It was found that 71% of cases are treated at teaching hospitals presumably due to the complexity and emergent nature of the disease. Furthermore, patients are found to have lengthier hospital stays, more surgeries, and a total cost of stay. Patients treated in hospitals who treated more Fournier’s gangrene cases showed a dramatic decrease in mortality by up to 80% in comparison to hospitals treating one case a year.^6^

Though difficult to diagnose in its early stages, prompt identification is important because early aggressive surgical debridement and antibiotic therapy have been shown to reduce mortality.^12^ This early recognition requires a high index of clinical suspicion. Broad antibiotic therapy addresses both gram positive and gram-negative organisms. Current recommendations include empiric treatment with broad spectrum antibiotics such as a carbapenem or Piperacillin-Tazobactam in addition to Vancomycin and Clindamycin.^13^ The use of Clindamycin is often recommended in infections with *S. aureus* and toxin-producing streptococci due to studies in animal models^14^indicating that sub-therapeutic levels can decrease toxin production by group A streptococci in necrotizing fasciitis. An observational study comparing conservative treatment (waiting for established Fournier’s) versus urgent exploration has demonstrated a shortened hospital stay, decreased numbers of operating room debridement sessions, and improvement in clinical outcome when early surgical exploration is performed.^15^ Given the importance of early diagnosis, tools such as the LRINEC score have been developed to help differentiate necrotizing infections from other soft tissue infections. These tools however are general guidelines and are limited by demonstration of a high number of false positives and false negatives.^16^

In our case, we present a previously healthy 64-year-old male with unclear medical history presenting with late-stage skin findings of Fournier’s gangrene. Because he was relatively without primary care and follow-up, records only indicated a risk factor of tobacco use. On further workup, he was found to have nodules on chest CT signifying possible metastatic disease and a 7cm mass from the anal verge, noted on exam. Biopsy reports of the rectal mass later showed invasive adenocarcinoma of colorectal origin. Although rare, research has shown some association between rectal cancer and Fournier’s gangrene.^17^,^18^,^19^ This specific patient’s malignancy likely caused his symptoms over the preceding 2 months before serving as a nidus for his more acute progression to Fournier’s gangrene just prior to presentation. His hospital course was overall comparatively quick given his advanced findings, and he was discharged home stable on day 9. This case highlights the importance of early identification for Fournier’s gangrene because early management is key to decreasing mortality. Current literature is robust, though we hope that updated and new images can help to further strengthen clinical judgment and decision-making in the Emergency room setting.

## Supplementary Information








