# Supplementary figures and images for: A Case Report of Fournier’s Gangrene
Source: J Educ Teach Emerg Med. 2022 Apr 15;7(2):V4–8. doi: 10.21980/J8Z356 (PMC10332744; doi:10.21980/J8Z356)

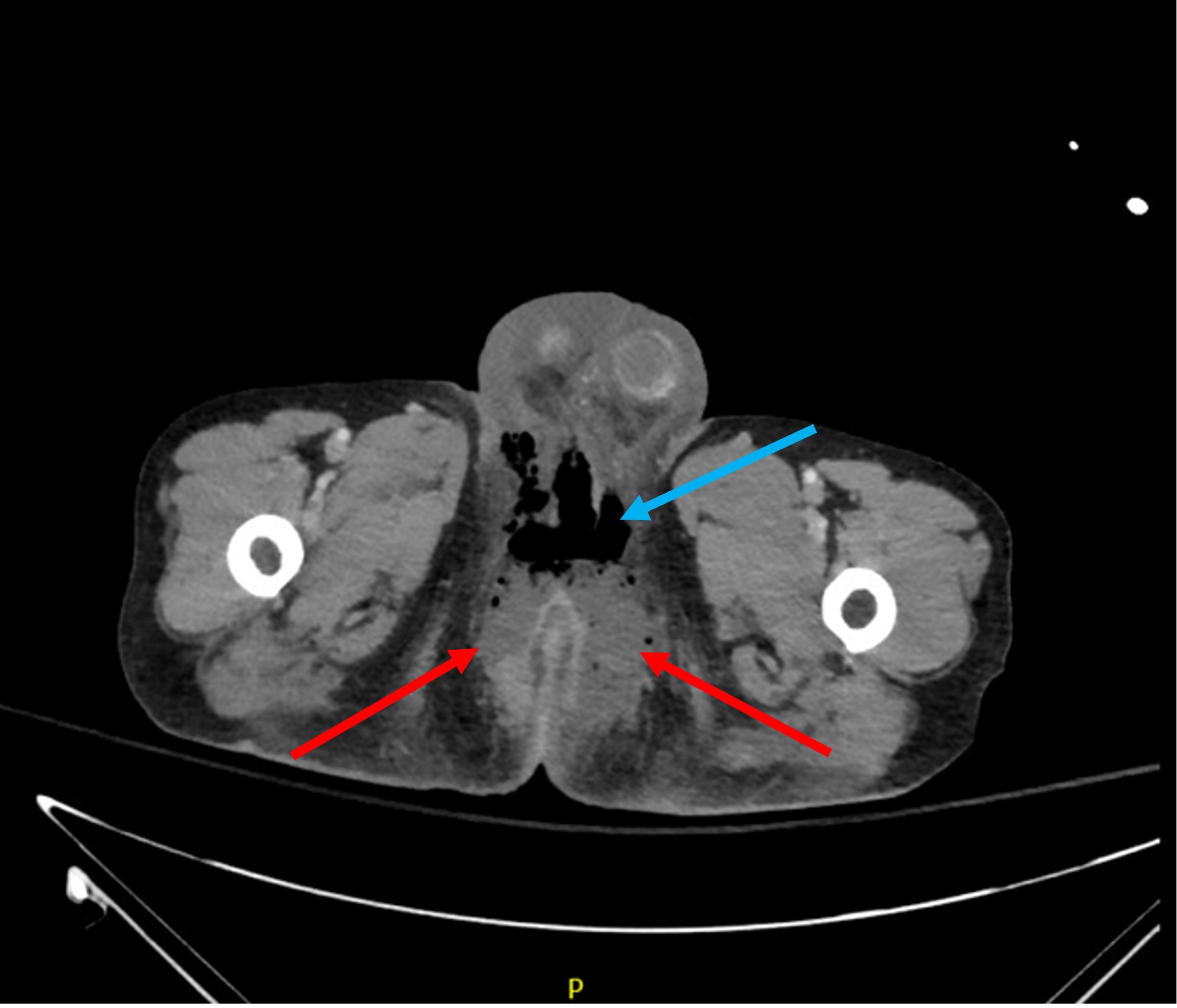

Supplement: Supplementary file 1 [file JETem-7-2-V4-supp1.jpg]

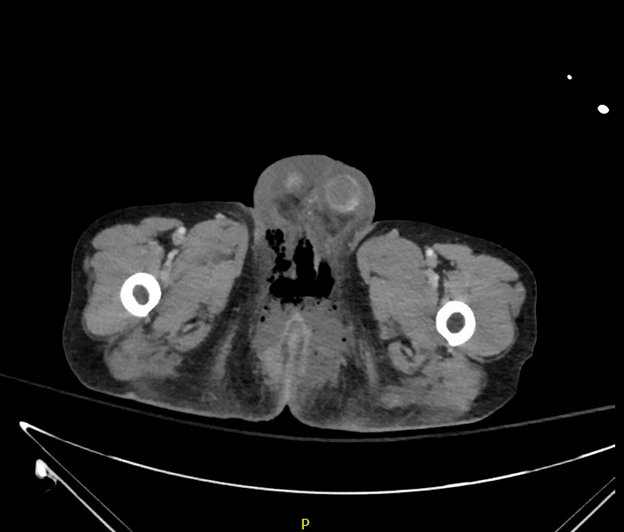

Supplement: Supplementary file 2 [file JETem-7-2-V4-supp2.jpg]

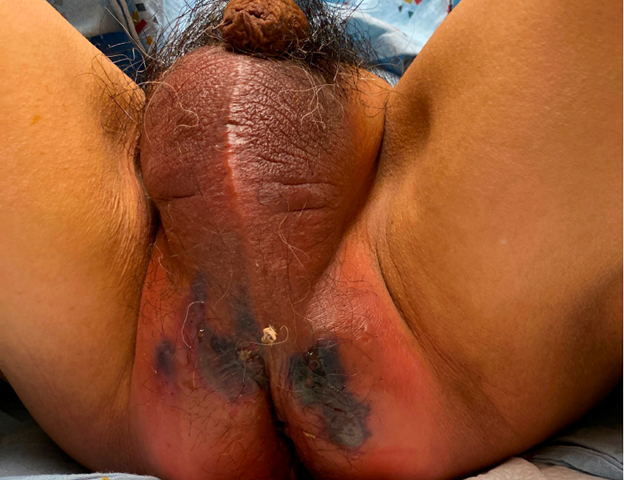

Supplement: Supplementary file 3 [file JETem-7-2-V4-supp3.jpg]

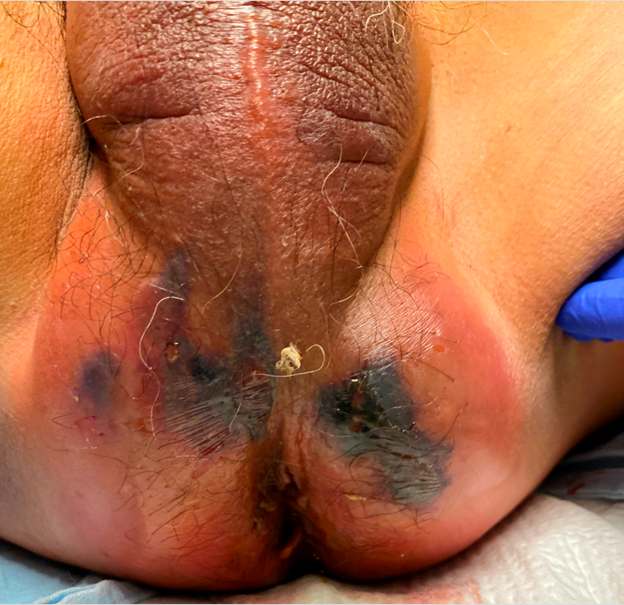

Supplement: Supplementary file 4 [file JETem-7-2-V4-supp4.jpg]
